# Peripherally derived myeloid cells induce disease-dependent phenotypic changes in microglia

**DOI:** 10.3389/fncel.2023.1295840

**Published:** 2023-12-14

**Authors:** Estrid Thougaard, Brianna Carney, Agnieszka Wlodarczyk, Roberta Brambilla, Kate Lykke Lambertsen

**Affiliations:** ^1^Neurobiology Research, Department of Molecular Medicine, University of Southern Denmark, Odense, Denmark; ^2^BRIDGE - Brain Research - Inter Disciplinary Guided Excellence, Department of Clinical Research, University of Southern Denmark, Odense, Denmark; ^3^The Miami Project to Cure Paralysis, Department of Neurological Surgery, University of Miami Miller School of Medicine, Miami, FL, United States; ^4^Department of Neurology, Odense University Hospital, Odense, Denmark

**Keywords:** myeloid cells, microglia, multiple sclerosis, ischemic stroke, neuroinflammation

## Abstract

In central nervous system (CNS) injury and disease, peripherally derived myeloid cells infiltrate the CNS parenchyma and interact with resident cells, propagating the neuroinflammatory response. Because peripheral myeloid populations differ profoundly depending on the type and phase of injury, their crosstalk with CNS resident cells, particularly microglia, will lead to different functional outcomes. Thus, understanding how peripheral myeloid cells affect the phenotype and function of microglia in different disease conditions and phases may lead to a better understanding of disease-specific targetable pathways for neuroprotection and neurorepair. To this end, we set out to develop an *in vitro* system to investigate the communication between peripheral myeloid cells and microglia, with the goal of uncovering potential differences due to disease type and timing. We isolated peripheral myeloid cells from mice undergoing experimental autoimmune encephalomyelitis (EAE), a model of multiple sclerosis, or acute cerebral ischemia by permanent middle cerebral artery occlusion (pMCAO) at different times after disease and probed their ability to change the phenotype of primary microglia isolated from the brain of adult mice. We identified changes not only dependent on the disease model, but also on the timepoint after disease onset from which the myeloid cells were isolated. Peripheral myeloid cells from acute EAE induced morphological changes in microglia, followed by increases in expression of genes involved in inflammatory signaling. Conversely, it was the peripheral myeloid cells from the chronic phase of pMCAO that induced gene expression changes in genes involved in inflammatory signaling and phagocytosis, which was not followed by a change in morphology. This underscores the importance of understanding the role of infiltrating myeloid cells in different disease contexts and phases. Furthermore, we showed that our assay is a valuable tool for investigating myeloid cell interactions in a range of CNS neuroinflammatory conditions.

## 1 Introduction

Diseases of the central nervous system (CNS) are etiopathologically diverse, ranging from acute disorders such as traumatic brain injury and stroke, to chronic neurodegenerative diseases like multiple sclerosis (MS) and Alzheimer’s. Because an immune-inflammatory response is common to virtually all of them (Lucas et al., 2006), targeting specific immune processes has been pursued for the treatment of neurological diseases (Correale et al., 2017; Lambertsen et al., 2019; Ruiz et al., 2019; Iadecola et al., 2020).

Cells of the myeloid lineage play an important role in the response to CNS injury and disease (Brendecke and Prinz, 2015). Microglia are the CNS-resident myeloid cells and are key regulators of neural homeostasis (Nayak et al., 2014). They are also a crucial component of the response to injury/disease and, in disease states, they undergo profound phenotypic changes signaling their activation. These include morphological changes, such as the transition to an amoeboid shape, and transcriptional changes that lead to increased expression and release of cytokines and chemokines (DiSabato et al., 2016; Becher et al., 2017). The advent of single cell omics has made it clear that microglial responses are highly heterogeneous and vary in time and space depending on the type, severity, and localization of injury/disease within the CNS. This has led to the identification of multiple coexisting microglial phenotypes with distinct and often opposite functions (Zheng et al., 2022; Vainchtein et al., 2023).

In MS, microglia can have both detrimental and protective effects (Rawji and Yong, 2013; Guerrero and Sicotte, 2020; Plastini et al., 2020). They are involved in demyelination (Zrzavy et al., 2017; Savarin et al., 2018), and in the experimental autoimmune encephalomyelitis (EAE) model, the early depletion of microglia or reduction of their activation improves the outcome (Goldmann et al., 2013; Nissen et al., 2018). Microglia can also be protective in EAE through production of anti-inflammatory cytokines and clearance of myelin debris (Kocur et al., 2015; Wlodarczyk et al., 2015). Depletion of microglia at late timepoints after EAE exacerbates inflammation and demyelination (Tanabe et al., 2019), indicating a finely tuned microglial function that differs according to the disease phase.

In acute neurological disease, such as ischemic stroke, microglia are the first cells to be activated and, as in MS, they have dual functions (Ma et al., 2017; Qin et al., 2019). Microglia release pro-inflammatory cytokines after experimental stroke, leading to disruption of the blood-brain-barrier (Clausen et al., 2008; Jolivel et al., 2015), synapse elimination, and neuronal death (Neher et al., 2013; Shi et al., 2021). Reducing the number of activated microglia increases the number of new neurons and improves functional outcome (Liu et al., 2007). Microglia can also limit the ischemic damage by reducing the size of the ischemic lesion, thus improving the outcome (Lalancette-Hébert et al., 2007; Lambertsen et al., 2009; Szalay et al., 2016), and the phagocytic properties of microglia are important in the recovery post-stroke (Kawabori et al., 2015).

Peripheral immune cells of the myeloid lineage are the first to infiltrate the CNS in neurological disease. Neutrophils are present in the inflamed areas shortly after the onset of ischemic stroke and EAE (Lewis et al., 2014; Beuker et al., 2021) and have been proposed as a biomarker for MS (Bisgaard et al., 2017). Macrophages also infiltrate the ischemic brain in large numbers early after stroke (Beuker et al., 2021), and in EAE, where T-cells are required for the induction of disease, monocytes were shown to be necessary for disease progression (Ajami et al., 2011). When participating in the neuroinflammatory response, peripheral myeloid cells carry out many of the same functions as their CNS-resident counterpart, microglia. However, their disease-induced phenotypic changes may occur with different timing and localization, leading to distinct and sometimes opposing functions (Ajami et al., 2011; Giles et al., 2018; Werner et al., 2020; Gao et al., 2017, 2023). Peripherally derived myeloid cells interact with CNS cells driving the acute neuroinflammatory response but can also persist long-term in the CNS parenchyma participating in the chronic neuroinflammatory response (Garcia-Bonilla et al., 2016), where they sustain various processes (Brendecke and Prinz, 2015; Giles et al., 2018; Lambertsen et al., 2019).

Because of the co-existence of microglia and peripherally derived myeloid cells in CNS disease, it is important to understand how their cross-talk might affect the outcome in different CNS pathologies (degenerative vs. traumatic) and disease stages (acute vs. chronic). The complexity and plurality of CNS cellular networks make it challenging to study discrete cell-cell interactions *in vivo*, and even more so under inflammatory conditions. A further obstacle is the functional and transcriptional similarities of the two populations, making it difficult to distinguish them *in vivo*. To overcome these challenges, we developed an *in vitro* system to investigate the communication between peripheral myeloid cells and microglia, with the goal of uncovering potential differences due to disease type and timing.

## 2 Materials and methods

### 2.1 Animals

Adult female C57Bl/6 mice (2 months old) were used for this study. The animals were kept in group housing under controlled temperature and humidity, a 12/12-h light/dark cycle, and with food and water *ad libitum*. The experiments carried out at the Animal Core Facility of The Miami Project to Cure Paralysis were performed according to protocols and guidelines approved by the Institutional Animal Care and Use Committee of the University of Miami. The experiments conducted at the Animal Facility of the University of Southern Denmark were performed in accordance with approved permits (J. no. 2019-15-0201-01620).

### 2.2 Permanent middle cerebral artery occlusion

The distal part of the left middle cerebral artery (MCA) was permanently coagulated to induce an experimental stroke, as previously described (Lambertsen et al., 2009; Yli-Karjanmaa et al., 2019). The surgery was performed under anesthesia with a mix of Hypnorm [fentanyl citrate (0.135 mg/mL, VetaPharma) and fluanisone (10 mg/mL, VetaPharma)], Midazolam (5 mg/mL, Hameln), and sterile water in the ratio 1:1:2. When the mouse was fully sedated, the skin was incised from the eye to the ear on the left side, the muscles were dissected to expose the skull, and a small hole was drilled above the MCA. The MCA was coagulated by pinching it with bipolar forceps and administering an electric current, hereby inducing a permanent middle cerebral artery occlusion (pMCAO). Mice were given subcutaneous injections of analgesics [Temgesic (0.001/20 mg body weight buprenorphine; Indivior)] every 8 h for 24 h, starting at the time of surgery, while being kept in a temperature-controlled heating cabinet. Eyes were coated in Viscotears ointment (2 mg/g, Bausch and Lomb) to prevent dehydration. The mice were euthanized at acute disease (1 day post-injury, dpi) or chronic disease (14 dpi) for isolation of peripheral myeloid cells, using an intraperitoneal injection of 0.2 mL pentobarbital (200 mg/mL) containing lidocaine (10 mg/mL) followed by transcardial perfusion with 20 ml phosphate-buffered saline (PBS).

### 2.3 Experimental autoimmune encephalomyelitis

Mice were induced with experimental autoimmune encephalomyelitis (EAE) following a protocol well established in the laboratory (Brambilla et al., 2011; Madsen et al., 2020). Briefly, on day 0 and 2, pertussis toxin (PTX, List Biological Laboratories, #181) diluted in PBS was injected intraperitoneally (500 μg/mouse). On day 1, myelin oligodendrocyte glycoprotein peptide 35–55 (MOG_35–55_, Bio-Synthesis, #A9349-1) emulsified in complete Freund’s adjuvant was injected subcutaneously (0.33 mg/mouse). Disease development was tracked daily, and locomotor dysfunction was scored on a standard 0–6 scale where 0 = no symptoms; 1 = loss of tail tone; 2 = fully flaccid tail; 3 = complete hind limb paralysis; 4 = complete forelimb paralysis; 5 = moribund; 6 = dead. Mice were euthanized at acute disease (20 days post-induction, dpi) or chronic disease (30 dpi) for isolation of peripheral myeloid cells. Inclusion criterion for the acute timepoint was presence of symptoms for a maximum of 7 days. Inclusion criterion for the chronic timepoint was presence of symptoms for a minimum of 17 days.

### 2.4 Primary microglia cultures

Primary microglia cultures were generated from whole brains of naïve adult (2 months old) C57Bl/6 mice. Following transcardial perfusion with PBS, brains were dissected out and enzymatically dissociated into a single-cell suspension using Neural Tissue Dissociation Kit (P) (Miltenyi Biotec, #130-092-628) followed by myelin removal with debris removal solution (Miltenyi Biotec, #130-109-398). Microglia were then isolated by magnetic-activated cell sorting (MACS) with LS columns (Miltenyi Biotec, #130-042-401) after incubation with anti-CD11b conjugated magnetic microbeads (Miltenyi Biotec, #130-093-634) according to the manufacturer’s protocol. Cells were seeded on poly-D-lysine coated plates (Sigma-Aldrich, #P7280) and maintained in complete medium consisting of RPMI 1640 Medium (Gibco, A1049101) supplemented with 20% L929 fibroblast conditioned media, 10% fetal bovine serum (FBS) (GEMINI, #900-108), 50 μM beta-mercaptoethanol, and 1% antibiotic-antimycotic (Gibco, #15240-062). Cells were plated in 24-well plates at a density of 37,000 cells/cm^2^ for RNA isolation, and in 24-well plates (indirect co-cultures) or 96-well plates (direct co-cultures) at a density of 16,000 cells/cm^2^ for morphological analysis. Microglia were cultured for 5 days with a partial media change (50%) every other day.

The primary microglia culture was assessed for purity by immunohistochemical staining and cell counting. Paraformaldehyde (PFA) fixed cells were incubated with goat anti-Iba1 (1:200, Novus Biologicals, #NB100-1028), rat anti-glial fibrillary acidic protein (GFAP) (1:500, Invitrogen, #13-0300) and rabbit anti-oligodendrocyte transcription factor 2 (Olig2) (1:200, Sigma-Aldrich, #AB9610) overnight at 4°C, followed by washing and subsequent 1-h incubation with secondary antibodies (1:750, Thermo Fisher, Donkey anti-goat 594 #A-11058; Donkey anti-rat 488 #A-21208; Donkey anti-rabbit 647 #A-31573). The cells were washed, and nuclei stained with 4′,6-diamidino-2-phenylindole (DAPI) (1:6000, Invitrogen, #D1306). Seventeen (17) randomly selected fields of view from 6 individual cell culture wells were imaged at 20X magnification, and the percentage of Iba1^+^ microglia, GFAP^+^ astrocytes, Olig2^+^ oligodendrocytes and Iba1^–^GFAP^–^Olig2^–^ cells out of the total number of DAPI^+^ cells were estimated using ImageJ software.

### 2.5 Peripheral myeloid cell isolation

Since secondary lymphoid organs, such as the spleen, coordinate the immune response in immune-mediated pathologies such as MS and EAE, (Zhu et al., 2007), while in ischemic stroke the direct output of myeloid cells from the bone marrow drives the peripheral innate immune response (Courties et al., 2015), we isolated myeloid cells from the spleen of EAE induced mice and from the bone marrow of pMCAO injured mice at acute and chronic disease phases. After perfusion with PBS, spleen or bone marrow was isolated and manually dissociated into single cell suspensions followed by red blood cell lysis. Myeloid cells were isolated by magnetic bead cell separation with anti-F4/80 MicroBeads (Miltenyi Biotec, #130-110-443) and LS columns (Miltenyi Biotec, #130-042-401) according to the manufacturer’s protocol. For indirect co-cultures, 100,000 myeloid cells in 1 ml media (density 100 cells/μl) were plated in cell culture inserts (cellQART 24-well Cell Culture Insert, Sterlitech, #9320402), which were then placed on top of naïve microglia that were maintained in culture for 5 days. For direct co-cultures, myeloid cells were labeled with carboxyfluorescein succinimidyl ester (CFSE) (2 μM dilution in PBS, BioLegend, #423801) for 10 min at 37°C. Then 50,000 myeloid cells in 500 μl media were plated in the 24-well plates and 10,000 myeloid cells in 100 μl media were plated in the 96-well plates (density 100 cells/μl) directly on top of naïve microglia maintained in culture for 5 days. Naïve unstimulated microglia without myeloid cells were used as controls. Three-five (3–5) biological replicates/condition with 2–6 technical replicates for gene expression analysis and 1–8 technical replicates for morphological analysis were plated.

### 2.6 High content analysis of cell morphology

After 24 h in co-culture, microglia (either alone or mixed with peripheral myeloid cells) were fixed with 4% PFA, washed, and stained with CellMask Deep Red Plasma Membrane Stain (1:5000, Invitrogen, #C10046) and DAPI (1:2000, Invitrogen, #D1306). Cells were imaged with the Opera Phenix Plus High Content Screening System (Perkin Elmer), and micrographs were analyzed with Harmony^®^ software package. Multiple filters were applied to eliminate artifacts using empirically determined cutoffs. For the purpose of this study, we extracted the following parameters: area, perimeter, roundness, length/width ratio, and percentage of elongated cells (with length/width ratio above 3). Within each well, multiple fields of view were automatedly sampled, and the average value per well was calculated for each parameter. For direct co-cultures, the algorithm was instructed not to include CFSE-labeled cells (peripheral myeloid cells) in the analysis. Morphological data were calculated as the mean of 1–8 technical replicates per mouse. To account for variability between cultures, the results were normalized to internal unstimulated microglia controls, before being calculated as fold change of the acute timepoint with direct exposure.

### 2.7 Collection of microglia for RNA extraction

Direct co-cultures underwent fluorescence-activated cell sorting (FACS) to separate microglia from peripheral myeloid cells after 24 h in co-culture. Briefly, cells were lifted using a cell scraper, spun down, and resuspended in DMEM-F12 (Gibco, #11320-033) supplemented with 5% FBS (GEMINI, #900-108). Cell suspensions were passed through a strainer to ensure single cell separation, and DAPI was added to label dead cells (NucBlue Fixed Cell Stain ReadyProbes reagent, Invitrogen, #R37606). Live DAPI^–^CFSE^–^ microglia were sorted directly into RNA extraction buffer from the Arcturus PicoPure RNA Isolation Kit (Applied Biosystems, #12204-01). Microglia from indirect co-cultures after 24 h in co-culture and unstimulated microglia controls were collected as described above and immediately resuspended in RNA extraction buffer from the Arcturus PicoPure RNA Isolation Kit. In all experiments, 2–6 technical replicates were pooled to obtain 3–5 samples/condition.

### 2.8 Gene expression analysis

Microglial RNA was isolated with the Arcturus PicoPure RNA Isolation Kit (Applied Biosystems, #12204-01). RNA concentration and purity were measured using a 2100 Agilent Bioanalyzer. RNA was reverse-transcribed to cDNA with the Sensiscript RT Kit (Qiagen, #205211) together with random primers (Promega, #C1181). Semiquantitative RT-PCR was performed with PowerUp SYBR Green Master Mix (Applied Biosystems, #A25742) and specific primers for the genes of interest (Table 1), according to the manufacturer’s instructions. cDNA samples from the pMCAO co-cultures underwent pre-amplification (14 cycles at 57°C) due to low concentrations. The PCR data were normalized to *Gapdh* expression before being calculated as fold change of unstimulated naïve microglia using the deltadeltaCt method.

**TABLE 1:** Primers used for RT-PCR.

Gene	Forward primer	Reverse primer
*Ccr6*	5′GGT GCA GGC CCA GAA CTC CA	5′TGC AGC TCC GGC CCA CTT TG
*Cd40*	5′CTA TGG GGC TGC TTG TTG AC	5′CCA TCG TGG AGG TAC TGT TT
*Cmklr1*	5′CTG GTG GTG ATC TAC AGC TT	5′ACA GTG TTC ACG GTC TTC TT
*Cxcr3*	5′CTC CTC TTC TTG CTG GGG CTG CTA	5′GAA GGT GTC CGT GCT GCT CA
*Gapdh*	5′GAG GCC GGT GCT GAG TAT GTC GTG	5′TCG GCA GAA GGG GCG GAG ATG A
*Il1b*	5′CTT CAA ATC TCA CAG CAG CAC ATC	5′CCA CGG GAA AGA CAC AGG TAG
*Lrp1*	5′GCG GTG TGA CAA CGA CAA T	5′GCA CTT GAA CTG GGT ACT GG
*Mertk*	5′AGA CTC CCA GTC AAC CAC AG	5′CAG GAG GTA GGA GCT TTG AT
*Tnf*	5′AGG CAC TCC CCC AAA AGA TG	5′TCA CCC CGA AGT TCA GTA GAC AGA
*Tnfrsf1a*	5′GCC CGA AGT CTA CTC CAT CAT TTG	5′GGC TGG GGA GGG GGC TGG AGT TAG
*Tnfrsf1b*	5′GCC CAG CCA AAC TCC AAG CAT C	5′TCC TAA CAT CAG CAG ACC CAG TG
*Trem2*	5′CAG CCC TGT CCC AAG CCC TCA AC	5′CTC CTC ACC CAG CTG CCG ACA CC

### 2.9 Statistical analysis

All data are expressed as mean ± SEM of 3–5 biological replicates. Data were analyzed with unpaired two-tailed *t*-test, and differences between groups were considered statistically significant at *p*-values ≤ 0.05.

## 3 Results

### 3.1 *In vitro* assay to study the interaction between peripherally derived myeloid cells and microglia

**FIGURE 1:**
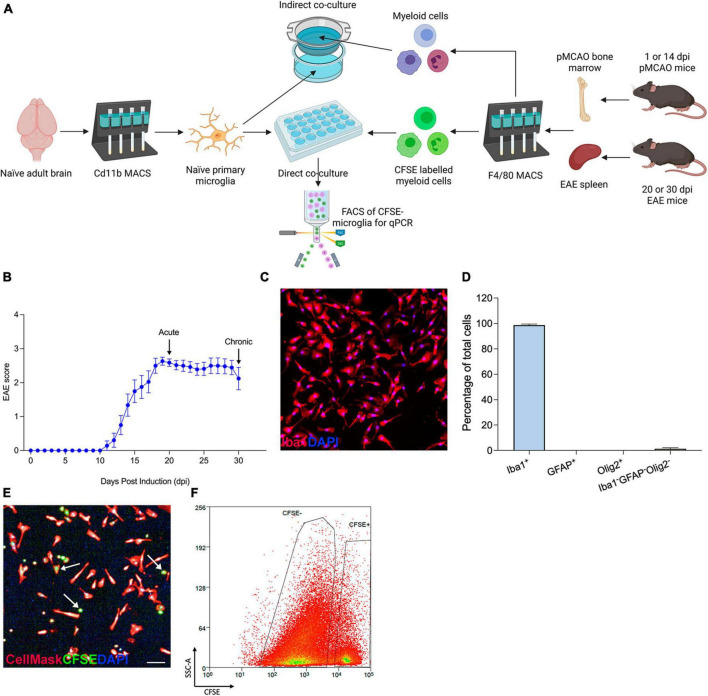
*In vitro* assay setup and validation. **(A)** Schematics of co-culture experiments. **(B)** Daily clinical scores of the EAE mice used in the present study. Data are shown as mean ± SEM, *n* = 14. **(C)** Representative 10X image of Iba1^+^DAPI^+^ naïve microglia. **(D)** Assessment of microglial purity by quantification of Iba1^+^ microglia, GFAP^+^ astrocytes, Olig2^+^ oligodendrocytes, and Iba1^–^GFAP^–^Olig2^–^ cells out of total DAPI^+^ cells. Data are expressed as mean ± SEM of 17 randomly selected areas imaged from 6 cell culture wells. **(E)** Representative image of a direct co-culture, with microglia stained with CellMask (red) and peripherally derived myeloid cells labeled with CFSE (green); scale bar = 100 μm. Arrows point at examples of CFSE-stained myeloid cells. **(F)** CSFE^–^ microglia isolated by FACS to exclude peripherally derived CSFE^+^ myeloid cells. Figure created with BioRender.com.

To investigate the cell-cell communication between myeloid cell populations in different disease conditions, we devised an *in vitro* assay in which peripherally derived myeloid cells isolated from mice undergoing EAE or cerebral ischemia (pMCAO) were co-cultured with microglia. This enabled us to study both direct communication requiring cell-to-cell contact and indirect communication driven by soluble factors between peripherally derived and CNS-resident myeloid cells. Adult microglia were cultured from naïve mice for 5 days and then exposed to peripherally derived myeloid cells (either from pMCAO or EAE mice) that were plated directly over the microglia monolayer or seeded in cell culture inserts (Figure 1A). In EAE, myeloid cells were obtained from the spleen at 20 dpi (the most acute phase of disease) and 30 dpi (during chronic disease) (Figure 1B). In pMCAO, myeloid cells were obtained from the bone marrow at 1 dpi (acute disease) and 14 dpi (chronic disease). The purity of adult primary microglia was confirmed by immunohistochemistry, with 99% of cells being positive for Iba1, and no contamination by GFAP^+^ astrocytes or Olig2^+^ oligodendrocytes (Figures 1C, D). In direct co-cultures (Figure 1E), to allow for discrimination of peripherally derived myeloid cells from microglia, myeloid cells were pre-labeled with CFSE and CFSE^–^ microglia were isolated by FACS (Figure 1F). Taken together, these validation experiments confirm that our *in vitro* assay works as intended and can be used to study different types of interactions between myeloid cell populations.

### 3.2 Peripherally derived myeloid cells induce disease-specific changes in microglial gene expression

**FIGURE 2:**
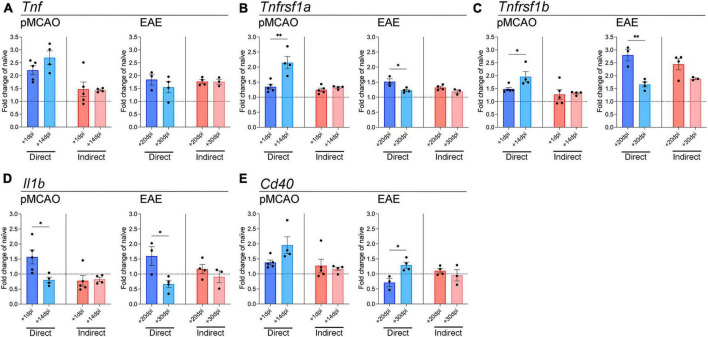
Gene expression analysis of pro-inflammatory cytokines and TNF receptor superfamily members in microglia. qPCR analysis of *Tnf* **(A)**, *Tnfrsf1a* **(B)**, *Tnfrsf1b* **(C)**, *Il1b* **(D)**, and *Cd40* **(E)** in adult microglia exposed to pMCAO or EAE myeloid cells. Direct: Direct contact and interaction between myeloid cells and microglia. Indirect: Indirect interaction between myeloid cells and microglia, only via soluble factors. Data are expressed as fold change of naïve microglia after normalization to *Gapdh* gene expression. The horizontal line at *y* = 1 indicates baseline expression in naïve microglia. Data are presented as mean ± SEM of 3–5 biological replicates. Each biological replicate is derived from 2–6 technical replicates. *p ≤ 0.05, **p ≤ 0.01, unpaired two-tailed *t*-test.

Myeloid cells respond to injury/disease by transitioning to an activated phenotype that is often characterized by upregulation of inflammatory genes such as cytokines and chemokines (Brendecke and Prinz, 2015; Becher et al., 2017). To investigate how peripheral myeloid cells in acute traumatic and chronic neurological disease may differ in their ability to drive microglial responses, we analyzed the expression of key inflammatory genes in microglia from our co-culture systems. Myeloid cells from both pMCAO and EAE mice increased microglial expression of *Tnf* compared to naïve unstimulated microglia, but no differences were observed between acute and chronic myeloid cell exposure (Figure 2A). Expression of TNF receptors *Tnfrsf1a* and *Tnfrsf1b* was also increased compared to baseline levels (Figures 2B, C). Direct contact with pMCAO myeloid cells induced higher expression of *Tnfrsf1a* and *Tnfrsf1b* when cells were obtained at the chronic timepoint, whereas direct contact with EAE myeloid cells induced lower expression of *Tnfrsf1a* and *Tnfrsf1b* when cells were obtained at the chronic timepoint. The highest expression of *Tnfrsf1a* was observed in pMCAO (chronic 14 dpi), and the highest expression of *Tnfrsf1b* was in EAE (acute 20 dpi) (Figures 2B, C). Interestingly, indirect interaction had minimal effect on microglial expression of these *Tnf* signaling genes, except for *Tnfrsf1b* which was elevated in both acute and chronic EAE (Figure 2C). For *Il1b*, direct contact with myeloid cells from acute disease (both pMCAO and EAE) increased microglial expression levels (Figure 2D). Finally, expression of *Cd40*, a member of the TNF superfamily whose ligand, CD40L, is important for microglial activation in inflammatory conditions (Ponomarev et al., 2006), was elevated by direct contact with chronic EAE myeloid cells compared to acute (Figure 2E). A similar trend (*p* = 0.065) was observed with pMCAO myeloid cells (Figure 2E).

**FIGURE 3:**
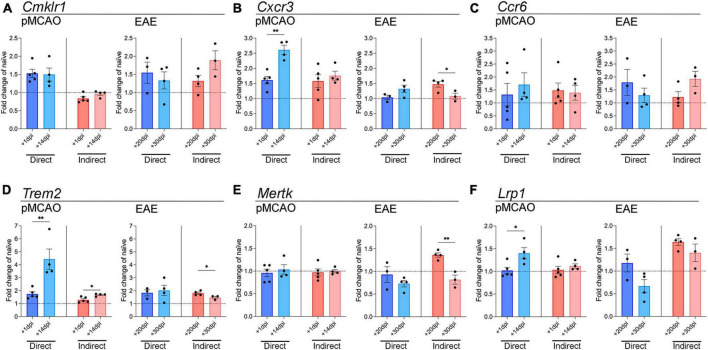
Gene expression analysis of chemokine receptor and phagocytosis genes in microglia. qPCR analysis of *Cmklr1* **(A)**, *Cxcr3* **(B)**, *Ccr6* **(C)**, *Trem2* **(D)**, *Mertk* **(E)**, and *Lrp1* **(F)** in adult microglia exposed to pMCAO or EAE myeloid cells. Direct: Direct contact and interaction between myeloid cells and microglia. Indirect: Indirect interaction between myeloid cells and microglia, only via soluble factors. Data are expressed as fold change of naïve microglia after normalization to *Gapdh* gene expression. The horizontal line at *y* = 1 indicates baseline expression in naïve microglia. Data are presented as mean ± SEM of 3–5 biological replicates. Each biological replicate is derived from 2–6 technical replicates. *p ≤ 0.05, **p ≤ 0.01, unpaired two-tailed *t*-test.

To look further into microglial inflammatory changes induced by myeloid cells, we examined genes involved in chemokine signaling, which are important for microglial migration toward sites of injury/disease (Becher et al., 2017; Ifergan and Miller, 2020). *Cmklr1*, the receptor for chemerin, was mildly elevated over baseline levels with EAE-derived cells at both timepoints, and with pMCAO-derived cells, but only with direct cell-cell interaction (Figure 3A). *Cxcr3* expression changed primarily with pMCAO myeloid cells in direct contact with microglia, showing a significant increase in chronic cells over acute (Figure 3B). With EAE-derived myeloid cells, *Cxcr3* changed minimally, showing a significant increase in acute over chronic cells after indirect cell-cell interaction (Figure 3B). No notable differences were found in *Ccr6* in any of the conditions (Figure 3C).

Next, we tested whether myeloid cells might affect the microglial phagocytic function. Phagocytosis is key in maintaining CNS homeostasis, and in disease states it is important to clear toxic cell debris (Fu et al., 2014). We examined the expression of *Trem2*, *Mertk*, and *Lrp1*, which have all been shown to be important for microglia phagocytic properties (Fu et al., 2014; Chuang et al., 2016; Shi et al., 2021). Myeloid cells from the chronic phase of pMCAO induced a higher expression of microglial *Trem2* compared to acute pMCAO cells, both with direct and indirect interaction (Figure 3D). Microglial *Trem2* expression with direct exposure to chronic pMCAO myeloid cells was the highest compared to naïve microglia (Figure 3D). Interestingly, *Trem2* changes with EAE myeloid cells were minimal. *Trem2* was mildly elevated over baseline levels in all conditions, and acute EAE myeloid cells induced higher expression than chronic EAE myeloid cells with indirect interaction (Figure 3D). A similar pattern with EAE myeloid cells was observed for *Mertk*, where expression was higher with acute than chronic cells after indirect interaction (Figure 3E). pMCAO myeloid cells did not induce any changes in *Mertk* expression (Figure 3E). Similar to *Trem2*, *Lrp1* was higher with chronic pMCAO myeloid cells compared to acute. EAE myeloid cells increased *Lrp1* expression after indirect contact, with no difference between acute and chronic (Figure 3F).

Collectively, these data illustrate that peripherally derived myeloid cells can induce transcriptional changes in naïve microglia, dependent on disease type and stage and mode of interaction (direct or indirect).

### 3.3 Peripheral myeloid cells from EAE mice induce changes in microglial morphology

**FIGURE 4:**
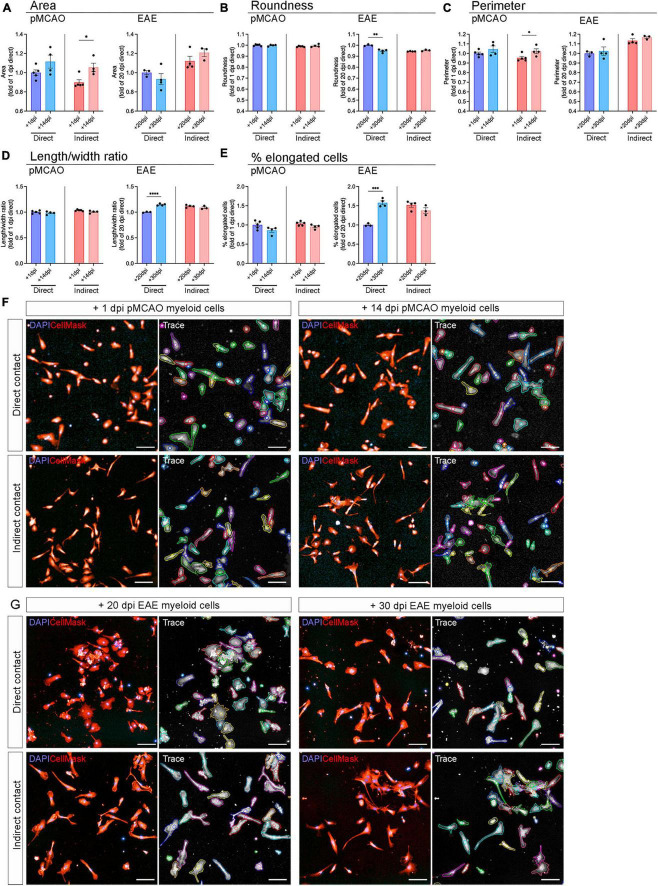
Microglial morphology after exposure to peripheral myeloid cells. Adult naïve microglia exposed to peripheral myeloid cells from pMCAO or EAE mice were evaluated for area **(A)**, roundness **(B)**, perimeter **(C)**, length/width ratio **(D)**, and percentage of elongated cells **(E)**. Data are normalized to naïve controls in each experiment and calculated as fold change of the acute timepoint with direct contact. Direct: Direct contact and interaction between myeloid cells and microglia. Indirect: Indirect interaction between myeloid cells and microglia, only via soluble factors. Data are presented as mean ± SEM of 3–5 biological replicates. Each biological replicate is the average of 1–8 technical replicates. *p ≤ 0.05, **p ≤ 0.01, ***p ≤ 0.001, ****p ≤ 0.0001, unpaired two-tailed *t*-test. Representative images of microglia exposed to pMCAO **(F)** and EAE **(G)** myeloid cells with tracing generated by Harmony^®^ high content analysis software. Scale bars = 100 μm.

Microglial activation driven by injury and disease is denoted by a series of phenotypic changes that include morphological adaptations whereby microglia transition from a ramified, branched phenotype to an ameboid phenotype with larger cell body and shorter processes (Woodburn et al., 2021). To further investigate whether myeloid cells changed microglial activation state, we evaluated microglial morphology using high content analysis, focusing on area, roundness, perimeter, length/width ratio, and quantifying the percentage of elongated cells. Overall, the morphological changes were relatively mild in all experimental conditions (Figures 4A–E). When exposed to myeloid cells from pMCAO mice, the most notable change was observed in cell size, where indirect cell interaction led to a reduced cell area (Figure 4A) and perimeter (Figure 4C) with acute myeloid cells, showing a significant difference compared to chronic. However, the microglia exposed to pMCAO myeloid cells appeared to have a similar morphology across conditions (Figure 4F). When exposed to myeloid cells from EAE mice, direct contact most notably affected microglial length/width ratio (Figure 4D) and % of elongated cells (Figure 4E), which decreased with acute interaction compared to chronic. These changes were evident as microglia became visibly more rounded (Figures 4B, G).

## 4 Discussion

Although microglia and infiltrating myeloid cells have previously been thought to act in similar ways in neurological disease, RNA sequencing studies revealed distinct microglia and myeloid cell populations with key differences in disease conditions (Lewis et al., 2014; Yamasaki et al., 2014; Guo et al., 2021; Zheng et al., 2022). Despite the considerable involvement of peripheral myeloid cells in neurological disease, we still lack a full understanding of how they drive different disease processes and affect CNS-resident cells. In the current study, we developed an *in vitro* assay to identify potential differences between acute traumatic and chronic neurodegenerative disease by investigating how peripheral myeloid cells from pMCAO or EAE mice affect naïve microglia. As the spleen coordinates the immune response in MS and EAE (Zhu et al., 2007) and cells from the bone marrow drive the immune response in ischemic stroke (Courties et al., 2015), we isolated myeloid cells from the spleen of EAE induced mice and from the bone marrow of pMCAO injured mice at acute and chronic disease phases. We found key differences in their ability to modulate gene expression and morphology of microglia, not only between the two disease models, but also through the course of disease. These changes were dependent on whether the cells were in direct contact or had interactions via soluble factors only. Peripheral myeloid cells increased microglial expression of most of the investigated genes compared to the baseline expression. This validates our *in vitro* system, as an increase in microglial gene expression is expected with exposure to myeloid cells derived from disease conditions.

We tested *Il1b* expression as it is released from microglia and infiltrating myeloid cells acutely in neuroinflammatory conditions and is involved in EAE progression and ischemic damage (Clausen et al., 2008; Yang et al., 2014; Lévesque et al., 2016). The finding that peripheral myeloid cells from the acute phase of both EAE and pMCAO mice induced higher *Il1b* expression in microglia compared to chronic phase myeloid cells suggests that activated peripherally derived myeloid cells might activate pathogen recognition receptors (PRRs) and inflammasome assembly acutely (Lee et al., 2013; Yang et al., 2014). Interestingly, even though microglial *Tnf* expression was increased upon interaction with peripheral myeloid cells from both disease models, no changes were seen between acute and chronic timepoints, indicating that differences between disease phases are not triggered by peripheral myeloid cells. Expression of microglial *Tnfrsf1a* and *Tnfrsf1b* was increased with direct exposure to peripheral myeloid cells from both pMCAO and EAE mice. In the acute phase of EAE, peripheral myeloid cells highly express TNF and other cytokines (Renno et al., 1995; Ifergan and Miller, 2020), which may cause the increased expression of microglial *Tnfrsf1a* and *Tnfrsf1b* after interaction with acute EAE peripheral myeloid cells in our experimental setting. In stroke, increased TNF receptor expression peak days after pMCAO induction (Lambertsen et al., 2007), supporting our finding that peripherally derived chronic pMCAO myeloid cells increase microglial TNF receptor expression to a larger extent than acute myeloid cells. It is noteworthy that peripherally derived myeloid cells in both disease models induced changes in TNFR1 and TNFR2 simultaneously, as these two receptors have distinct and opposing functions in neurological disease (Dong et al., 2016; Papazian et al., 2021). This suggests that peripheral myeloid cells can induce both detrimental and protective effects in the microglia.

CD40 is upregulated with microglial activation and increases the release of cytokines and chemokines (Benveniste et al., 2004; Plastini et al., 2020). CD40 has a major role in EAE progression (Hart et al., 2005). Indeed, full activation of microglia in EAE depends on CD40, and lack of CD40 leads to lower T-cell proliferation and less severe disease (Ponomarev et al., 2006). The increase in microglial *Cd40* expression with exposure to peripheral myeloid cells from chronic EAE indicates that myeloid cells can induce *Cd40* expression, which might drive disease progression in the chronic stage through increased microglial activation and possibly T-cell proliferation.

Peripheral myeloid cells might have a more detrimental phenotype at the chronic timepoint following pMCAO, for example by providing “eat-me” signals to microglia, which in turn upregulate the expression of genes involved in phagocytosis. Thus, the increases in *Trem2* and *Lrp1* in microglia exposed to peripherally derived chronic pMCAO myeloid cells could be protective by eliminating detrimental infiltrating myeloid cells (Neumann et al., 2008). This is supported by several studies reporting an important role of Trem2 and Lrp1 in phagocytosis and neuroprotection in neurological disease (Takahashi et al., 2005; Fernandez-Castaneda et al., 2013; Kawabori et al., 2015; Chuang et al., 2016). Increased phagocytosis may also be detrimental if the targets are viable neurons (Neher et al., 2013; Shi et al., 2021), leading to a worsening of the ischemic damage at the chronic stage. However, phagocytosis of viable neurons depends primarily on Mertk (Neher et al., 2013; Shi et al., 2021), which was not upregulated in microglia following exposure to pMCAO peripheral myeloid cells in our study. Future studies using metabolomics and/or lipidomics, as well as scRNAseq, would help identify and quantify pathways and networks of cellular lipids and metabolites that change in microglia following exposure to myeloid cells at acute and chronic time points after EAE and pMCAO.

RNA sequencing studies reveal that peripheral myeloid cells from EAE mice upregulate multiple soluble cytokines and chemokines, especially in the acute phase of disease (Lewis et al., 2014; Yamasaki et al., 2014; Gao et al., 2023). This may explain the upregulation in microglial *Trem2*, *Mertk*, and *Cxcr3* that we observed with indirect contact with peripheral myeloid cells from acute EAE. Additionally, several studies demonstrated that infiltrating monocytes have very different transcriptional profiles at onset and peak phases of EAE compared to the recovery phase (Yamasaki et al., 2014; Gao et al., 2023). This fits well with our data, where we found microglia interacting with peripheral myeloid cells from acute EAE to have higher gene expression than with myeloid cells from chronic EAE, with the exception of *Cd40*. Also it was the exposure to acute EAE peripheral myeloid that altered microglial morphology to become more rounded, which is a hallmark of microglial activation (Stence et al., 2001; Woodburn et al., 2021). Overall, peripherally derived myeloid cells from acute EAE appear to be the most effective at inducing microglia activation, leading to changes in both gene expression and morphology.

This study prioritized investigating changes in gene expression, as a measure of whether peripheral myeloid cells were able to induce changes in naïve microglia using our *in vitro* assay. Future studies should aim to investigate whether the changes observed in microglial gene expression, persist as changes in protein levels. Moreover, as microglia will be activated in disease conditions (Woodburn et al., 2021), repeating the study with microglia isolated from EAE and pMCAO mice, to study the effect of activated peripheral myeloid cells on activated microglia, should be persued. Using naïve microglia in the present study was prioritized, as it allowed us to study the specific effect of activated peripheral myeloid cells, without involvement from intrinsic microglial activation. Additionally, it would be interesting to reverse the assay, to study the effect of microglia derived from different disease conditions on naïve peripheral myeloid cells.

In conclusion, we developed an *in vitro* system that allowed us to investigate the communication between peripheral myeloid cells and microglia. We identified peripheral myeloid cell-induced changes in microglia not only dependent on the disease model, but also on the disease phase at which myeloid cells were isolated. This underscores that neuroprotective and neuroreparative therapies must be tailored to each condition, and no myeloid modulating approach fits all. Our assay can be used to identify potential targets in myeloid cells under specific disease conditions, opening up the possibility of modulating these *in vivo*, either genetically or pharmacologically, in future studies, to assess their specific role in disease development. Furthermore, we showed that our assay is a valuable tool for investigating myeloid cell interactions in a range of CNS neuroinflammatory conditions.

## Data availability statement

The raw data supporting the conclusions of this article will be made available by the authors upon reasonable request.

## Ethics statement

The animal study was approved by the Institutional Animal Care and Use Committee of the University of Miami and the Danish Animal Inspectorate (J. no. 2019-15-0201-01620). The study was conducted in accordance with the local legislation and institutional requirements.

## Author contributions

ET: Data curation, Formal analysis, Funding acquisition, Investigation, Methodology, Writing – original draft, Writing – review and editing. BC: Methodology, Writing – review and editing. AW: Data curation, Methodology, Writing – review and editing. RB: Conceptualization, Formal analysis, Funding acquisition, Resources, Supervision, Validation, Writing – review and editing. KLL: Conceptualization, Formal analysis, Funding acquisition, Project administration, Resources, Supervision, Validation, Writing – review and editing.
